# Cardiovascular magnetic resonance black-blood thrombus imaging for the diagnosis of acute deep vein thrombosis at 1.5 Tesla

**DOI:** 10.1186/s12968-018-0459-6

**Published:** 2018-06-25

**Authors:** Hanwei Chen, Xueping He, Guoxi Xie, Jianke Liang, Yufeng Ye, Wei Deng, Zhuonan He, Dexiang Liu, Debiao Li, Xin Liu, Zhaoyang Fan

**Affiliations:** 1 Department of Radiology, Guangzhou Panyu Central Hospital, Guangzhou, 511400 Guangdong China; 2 Medical Imaging Institute of Panyu, Guangzhou, 511400 Guangdong China; 3 The Sixth Affiliated Hospital, Guangzhou Medical University, Xinzao, Panyu District, Qingyuan, 511518 Guangdong China; 4 Department of Biomedical Engineering of Basic Medical School, Guangzhou Medical University, Guangzhou, 511436 Guangdong China; 5 Biomedical Imaging Research Institute, Cedars-Sinai Medical Center, Los Angeles, CA 90048 USA; 6 Lauterbur Research Center for Biomedical Imaging, Shenzhen Institutes of Advanced Technology, Chinese Academy of Sciences, Shenzhen, 518055 Guangdong China

**Keywords:** Magnetic resonance imaging, Deep vein thrombosis, Venous thrombosis, Venography

## Abstract

**Background:**

The aim was to investigate the feasibility of a cardiovascular magnetic resonance (CMR) black-blood thrombus imaging (BBTI) technique, based on delay alternating with nutation for tailored excitation black-blood preparation and a variable flip angle turbo-spin-echo readout, for the diagnosis of acute deep vein thrombosis (DVT) at 1.5 T.

**Methods:**

BBTI was conducted in 15 healthy subjects and 30 acute DVT patients. Contrast-enhanced CMR venography (CE-CMRV) was conducted for comparison and only performed in the patients. Apparent contrast-to-noise ratios between the thrombus and the muscle/lumen were calculated to determine whether BBTI could provide an adequate thrombus signal for diagnosis. Two blinded readers assessed the randomized BBTI images from all participants and made independent decisions on the presence or absence of thrombus at the segment level. Images obtained by CE-CMRV were also randomized and assessed by the two readers. Using the consensus CE-CMRV as a reference, the sensitivity, specificity, positive and negative predictive values, and accuracy of BBTI, as well as its diagnostic agreement with CE-CMRV, were calculated. Additionally, diagnostic confidence and interobserver diagnostic agreement were evaluated.

**Results:**

The thrombi in the acute phase exhibited iso- or hyperintense signals on the BBTI images. All the healthy subjects were correctly identified from the participants based on the segment level. The diagnostic confidence of BBTI was comparable to that of CE-CMRV (3.69 ± 0.52 vs. 3.70 ± 0.47). High overall sensitivity (95.2%), SP (98.6%), positive predictive value (96.0%), negative predictive value (98.3%), and accuracy (97.7%), as well as excellent diagnostic and interobserver agreements, were achieved using BBTI.

**Conclusion:**

BBTI is a reliable, contrast-free technique for the diagnosis of acute DVT at 1.5 T.

## Background

Deep vein thrombosis (DVT) is a common clinical disease that can lead to severe complications such as pulmonary embolism, post-thrombotic syndrome, venous ulcer, and chronic pulmonary artery hypertension [1–3]. Early detection of newly formed thrombus is important for timely thrombolytic therapy and minimizing the occurrence of life-threating pulmonary embolism [4–6].

Several medical imaging techniques have been developed for diagnosing DVT in recent decades. X-ray contrast venography has traditionally been the gold standard [7]; however, the need for iodinated contrast agent and radiation exposure, as well as the technique’s invasive nature, have led to its rare use in purely diagnostic settings [8]. Ultrasonography is currently a first-line technique used for the diagnosis in patients with clinically suspected DVT because of operation convenience, low cost, and high diagnostic sensitivity (94%) and specificity (98%) [9].

However, the disadvantages of ultrasound include operator dependence, limited visualization in the pelvis station, and an inability to differentiate the phases of thrombus [8, 10, 11] Cardiovascular magnetic resonance (CMR) imaging may serve as an alternative or complementary imaging tool to ultrasoiund [8, 12]. Previous studies have demonstrated that contrast-enhanced CMR venography (CE-CMRV) is reliable for assessing deep and superficial venous systems and has a high sensitivity (92%) and specificity (94.8%) for detecting DVT [13–15]. However, this technique may be contraindicated in patients with an allergy to gadolinium, and in pregnancy or severe renal dysfunction.

Other CMRV techniques that do not need contrast agent can also be used for the diagnosis of DVT. These techniques include time-of-flight, phase contrast, and balanced steady-state free precession (bSSFP), which all allow the indirect identification of thrombus because the stationary tissue shows a signal void within the venous lumen [16–18]. However, these techniques cannot directly visualize the thrombus signal, which is important for clinical decision-making in DVT treatment [19].

In 1997, Moody et al. introduced a contrast-free CMR technique called MR direct thrombus imaging (MRDTI) for the diagnosis of DVT [20]. The technique utilizes the short-T1 methemoglobin within the thrombus at certain phases (primarily acute and subacute) to generate a bright thrombus signal on T1-weighted images, thus allowing DVT to be readily identified [6]. However, owing to the low concentration in methemoglobin, hyperacute or chronic thrombi appear relatively dark on MRDTI, and their visualization can be confounded by the surrounding venous blood, potentially leading to an inaccurate estimation of DVT distribution. This necessitates a combination of the technique with MR venography when characterizing DVT progression [21].

Recently, three-dimensional (3D) T1-weighted black-blood CMR techniques have been developed for the diagnosis of DVT without the use of a contrast agent [22, 23]. The principle underlying the techniques is that venous blood flow signal is suppressed to allow the intra-luminal thrombus to be visualized within the venous lumen. The technique proposed by Treil et al. relies on the inherent black-blood effect of the 3D variable-flip-angle turbo spin-echo sequence, which may be inadequate for suppressing the signal from extremely slow venous blood flow [22]. An improved technique called black-blood thrombus imaging (BBTI) combines the same 3D turbo spin echo readout with an additional black-suppressing preparation, i.e., delay alternating with nutation for tailored excitation (DANTE), to achieve better DVT visualization [23]. A preliminary study demonstrated that BBTI can detect non-acute DVT at 3 Tesla (T) with high sensitivity (90.4%) and specificity (99.0%), using CE-CMRV as a reference.

The performance of BBTI for detecting acute (particularly hyperacute) DVT at a lower but more commonly used field strength (i.e., 1.5 T) remains unknown. A thrombus in the hyperacute phase is rich in deoxyhemoglobin rather than methemoglobin and can therefore have short T2 relaxation values [24, 25]. This may lead to lower signal intensity in acute DVT and reduced contrast between DVT and the surrounding venous blood and vessel wall. Lower signal-to-noise ratio (SNR) at 1.5 T may further impair the visualization of DVT. The purpose of this prospective study was to assess the image quality and diagnostic performance of BBTI at 1.5 T in patients with acute DVT, using CE-CMRV as the reference standard.

## Methods

### Subjects

Fifteen healthy subjects (38.1 ± 14.2 years, 7 women) with no history of peripheral vascular disease, and 30 acute DVT patients (54.1 ± 17.0 years, 22 women) were studied. The patients were consecutively enrolled from a local hospital from September 2015 to May 2017 (Table 1) and were all inpatients at the time of participation. One of the patients was first diagnosed with DVT 9 years ago and had a symptomatic recurrent ipsilateral DVT for 10 days. All the others were experiencing symptoms for the first time. The duration of symptoms was 7.3 ± 4.0 days (range, 2–14 days), and therefore all cases were deemed acute, according to the duration from symptom onset to CMR scan, as follows: acute phase (≤ 14 days), subacute phase (15–28 days), subacute-to-chronic phase (29–180 days), and chronic phase (> 180 days) [23, 26]. The initial diagnosis of DVT was made using the D-dimer test and ultrasound performed by experienced sonographers as part of routine standard of care. The ultrasound test only targeted the leg(s) with DVT symptoms, and the duration between the ultrasound test and CMR scan was less than 24 h. The exclusion criteria were known contraindications to CMR (e.g., claustrophobia, pregnancy, gadolinium allergy or renal failure). This prospective study was approved by the institutional review board, and written informed consent was obtained from all the subjects.

**Table 1:** Patient characteristics

Characteristics	
Age, mean ± SD (range), (years)	54.1 ± 17.0 (27–84)
Sex	8 male, 22 female
Body mass index	23.7 ± 1.4 (20.3–27.0)
Symptom duration, mean ± SD (range), (days)	7.3 ± 4.0 (2–14)
Symptom duration ≤7 days, n (%)	16 (53.3%)
Symptoms:	
Leg pain, n (%)	27 (90.0%)
Swollen leg, n (%)	30 (100%)
Local warmth, n (%)	24 (80.0%)
Recurrence (%), n (%)	1 (3.3%)
Pulmonary embolism, n (%)	4 (13.3%)
Recent trauma (< 1 month), n (%)	1 (3.3%)
Recent surgery (< 1 month), n (%)	6 (20.0%)
Previous venous thromboembolism, (pulmonary embolism or DVT), n (%)	1 (3.3%)

Note: *SD* standard deviation, *DVT* deep vein thrombosis

### CMR imaging

CMR imaging was performed on a 1.5 T scanner (MAGNETOM Avanto, Siemens Helathineers, Erlangen, Germany). BBTI was performed on both patients and healthy subjects. To scan the entire lower limb, each of the subjects was placed in the supine position (feet first) and underwent 3-station scanning using a 6-channel body coil and an 8-channel peripheral vascular coil, as well as integrated spine coils. The coverage of the first station was from the lower inferior vena cava to the proximal femoral vein, the second station from the femoral vein and deep femoral vein to the proximal popliteal vein, and the third station from the popliteal vein to the distal calf vein. The scan time of each station was 3–5 min. To evaluate the accuracy of BBTI for the diagnosis of DVT, CE-CMRV using a 3D gradient echo fast low-angle shot (FLASH) pulse sequence for data acquisition served as the reference standard and was only performed in the patients. Following a mask measurement, the FLASH sequence was initiated when contrast agent (gadopentetate dimeglumine [Magnevist; Bayer Pharmaceuticals, Leverkusen, Germany]) arrived at the iliac artery, as detected using the Care Bolus technique (Siemens Healthineers). A fixed dose of 30 mL (469 mg/mL) of contrast agent was administered intravenously at a speed of 3.0 mL/second in the median cubital vein using a remote-controlled injection system (Medrad Spectris, Indianola, Pennsylvania, USA). The contrast injection was followed by a 20 mL saline flush injected at the same rate. The scan was continuously repeated three times to capture a well-enhanced time frame. The scan parameters are presented in Table 2.

**Table 2:** Imaging parameters of black blood thrombus imaging (BBTI) and contrast-enhanced cardiovascular magnetic resonance venography (CE-CMRV)

	BBTI	CE-CMRV
Repetition time (ms)	650	4.37
Echo time (ms)	11	1.41
Turbo factor	40	NA
Fat suppression	yes	no
Flip angle (degree)	T1 variable	25
FOV read (mm)	352	500
Number of partitions	208–256	112
Voxel size (mm^3^)	1.4 × 1.4 × 1.4	1.5 × 1.1 × 1.2
Interpolated voxel size (mm^3^)	0.7 × 0.7 × 0.7	0.75 × 0.55 × 0.6
Bandwidth (Hz/pixel)	698	250
DANTE pulse train length	125	NA
DANTE flip angle	15	NA

Note: *FOV* field-of-view, *DANTE* delay alternating with nutation for tailored excitation, *NA* non applicable

### Image analysis

All the images were loaded onto a workstation (Leonardo, Siemens Healthineers) for post-processing and qualitative and quantitative evaluations by two readers (Y. Y. and W. D) with over 10 years’ of CMR experience.

Qualitative image analysis at the vessel segment level was performed on both healthy subjects and DVT patients. The venous system of each lower limb was divided into the inferior vena cava (IVC), common, internal and external iliac veins, common femoral vein, femoral vein, deep femoral vein, popliteal vein, tibiofibular trunk vein, anterior and posterior tibial veins, fibular vein, and the great and small saphenous veins on both sides. If vessel collateralization was identified, the collateral segments were also assessed. Collateralization is defined as the growth of new veins that serve the same vascular bed as the original veins, which cannot themselves adequately supply the vascular bed. As collateral segments are usually too small or complex to be correctly identified, only the clots that blocked the collateral segments that were dilated large enough to be identified on both the BBTI and CE-CMRV images were considered for analysis.

All the images obtained from the healthy subjects and patients were mixed and randomized in preparation for reading on a per-segment vessel basis by two independent readers for the absence or presence of thrombus, without knowledge of each subject’s information or the ultrasound test results. A time interval of 2 weeks was required for image review of BBTI and CE-CMRV to avoid memory bias. The image quality and diagnostic confidence of each observed venous segment in all the subjects were assessed on a 4-point scale (1: poor; 4: excellent) [22, 23]. If two readers disagreed with each other regarding the presence or absence of DVT on standard CE-MRV, the readers reached a consensus after checking all the available information, including the ultrasound results.

To illustrate that the thrombus in the acute phase had adequate signal intensity (SI) on the BBTI images for diagnosis, the apparent SNR of the thrombus, muscle, and the dark venous lumen were calculated as the mean SI divided by the noise (σ_n_), and the apparent contrast-to-noise ratio (CNR) between the thrombus and the muscle ([SI_thrombus_ – SI_muscle_]/σ_n_) and between the thrombus and the venous lumen ([SI_thrombus_ – SI_lumen_]/σ_n_) were also calculated. The noise was the standard deviation of the SI determined in four artefact-free background regions to minimize bias due to the inhomogeneous signal. SI was measured as the mean signal intensity within a manually drawn region of interest (ROI). Notably, the ROIs of the thrombus and dark lumen were determined in consensus by the two readers. If visually different SI of the thrombi (i.e., iso- or hyper-intense signal relative to the muscle) were observed in the same patient, the SI was measured separately for the SNR and CNR calculations.

The feasibility of using BBTI for thrombus volume measurement and understanding thrombus progression in the acute phase was explored. Specifically, thrombi in each patient were segmented in a semiautomatic fashion using VesselMass (Vessel Mass, Leiden University Medical Center, Leiden,,The Netherlands), and iso−/hyperintense thrombi were measured separately for each patient. The percentage of the iso-intense thrombus in the total thrombus volume was calculated and reported for each patient.

### Statistics

The image quality and diagnostic confidence scores of both healthy subjects and DVT patients were presented as the mean ± standard deviation (SD). Interobserver agreement using the kappa statistic was performed for the healthy subjects and patients in all the venous segments for the presence or absence of thrombus, image quality, and diagnostic confidence of BBTI.

The sensitivity, specificity, positive and negative predictive values, and accuracy of BBTI in the diagnosis of DVT patients were calculated using a standard 4 × 4 contingency table, with the consensus CE-CMRV as a reference. A paired sample Student’s *t* test was used to compare the continuous variables, and the data were presented as the mean ± SD. Interobserver agreement and agreement between BBTI and the consensus CE-CMRV were tested using Cohen’s κ test. Agreement was rated as fair (kappa value κ = 0.21–0.40), moderate (κ = 0.41–0.6), substantial (κ = 0.61–0.80), or excellent (κ > 0.80) [27]. Of note, *p* <  0.05 indicated statistical significance. Linear regression was used to demonstrate the trends of the thrombus signal variation along with the duration of symptom onset increase. The Statistical Package for the Social Sciences (SPSS 17.0, International Business Machines, Armonk, New York, USA) software was used for the statistical analysis.

## Results

**Fig. 1:**
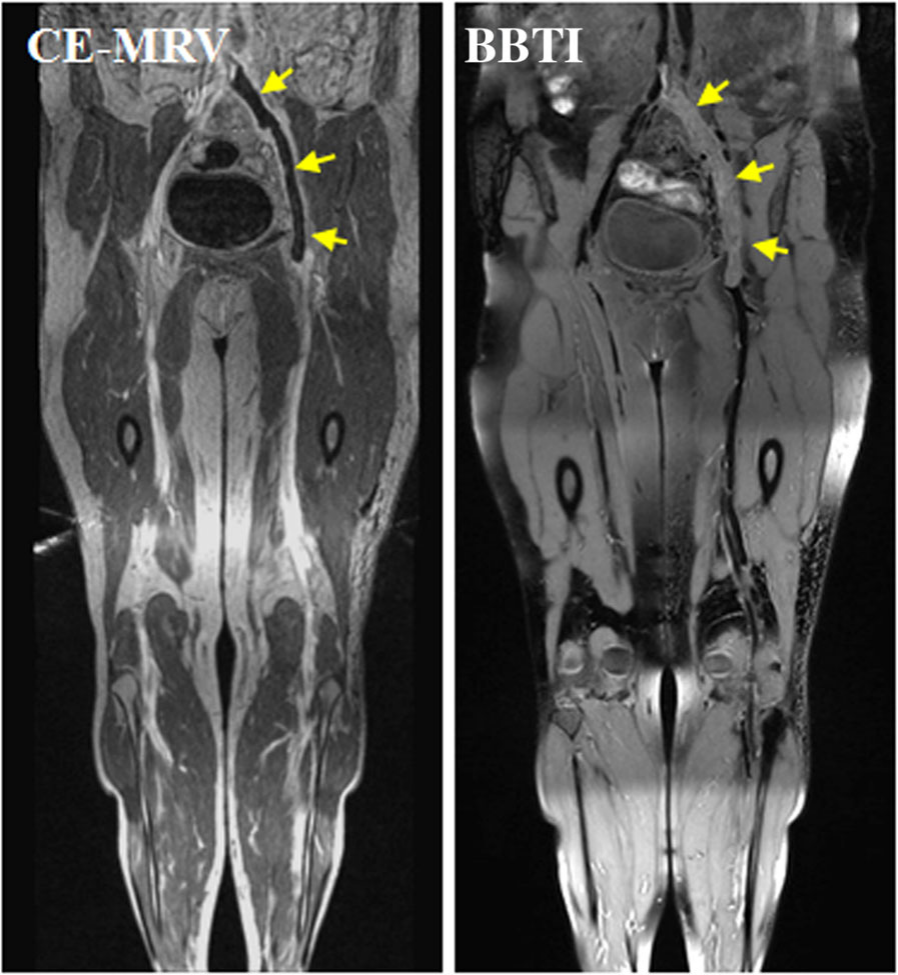
Representative images obtained by contrast-enhanced cardiovascular magnetic resonance (CE-CMRV) and black blood thrombus imaging (BBTI) from a 55-year-old woman with deep venous thrombosis (DVT) symptom onset at 5 days. The thrombus detected by BBTI showed iso-intense signals within the black-blood venous lumen. The locations and sizes of the thrombi between BBTI and CE-CMRV matched (yellow arrows)

**Fig. 2:**
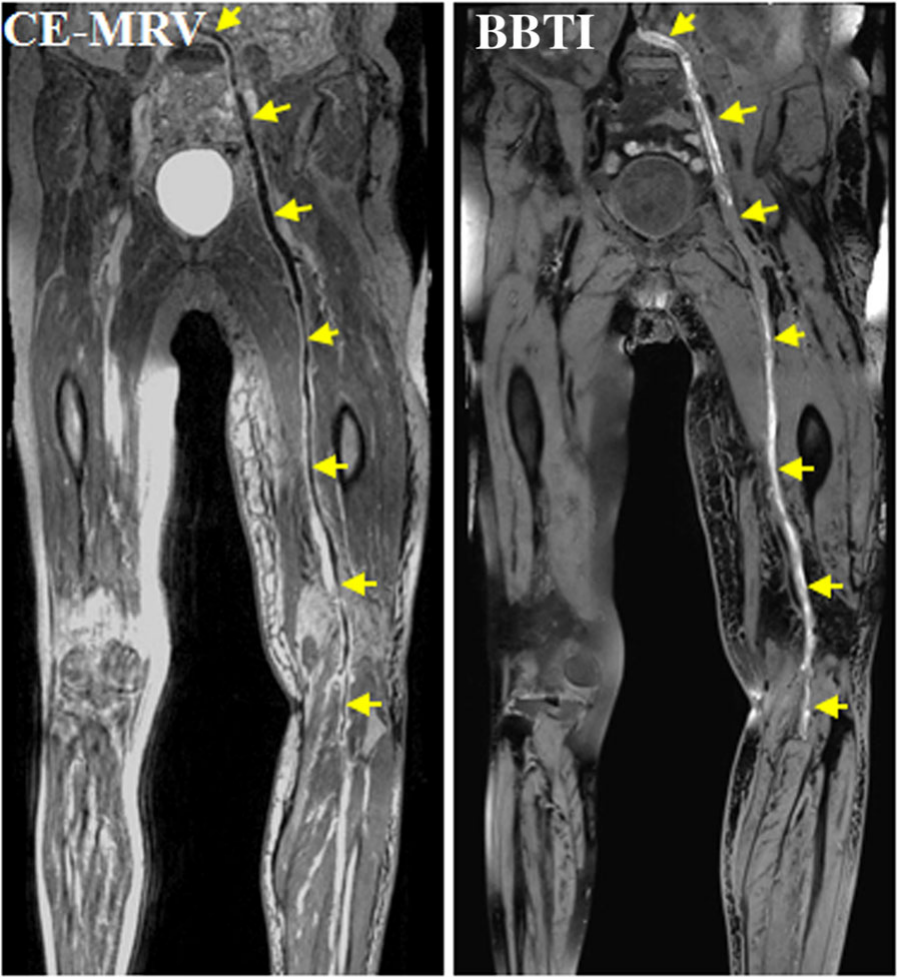
Representative images obtained by CE-CMRV and BBTI from a 68-year-old woman with DVT symptom onset at 8 days. The thrombus detected by BBTI showed iso- and hyperintense signals within the black-blood venous lumen. The locations and sizes of the thrombi between BBTI and CE-CMRV matched (yellow arrows)

**Fig. 3:**
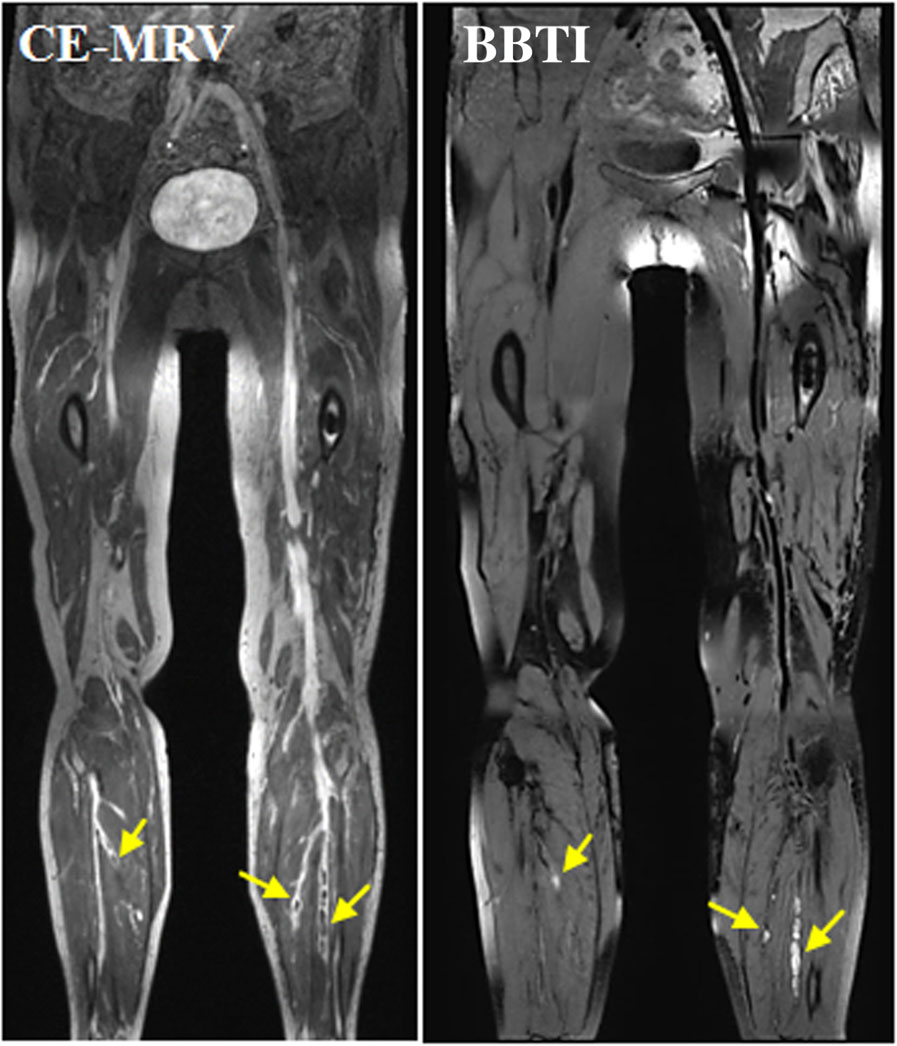
Representative images obtained by CE-CMRV and BBTI from a patient with DVT symptom onset at 10 days. The small thrombus can also be detected by BBTI and matched well with that seen with CE-CMRV (yellow arrows)

**Fig. 4:**
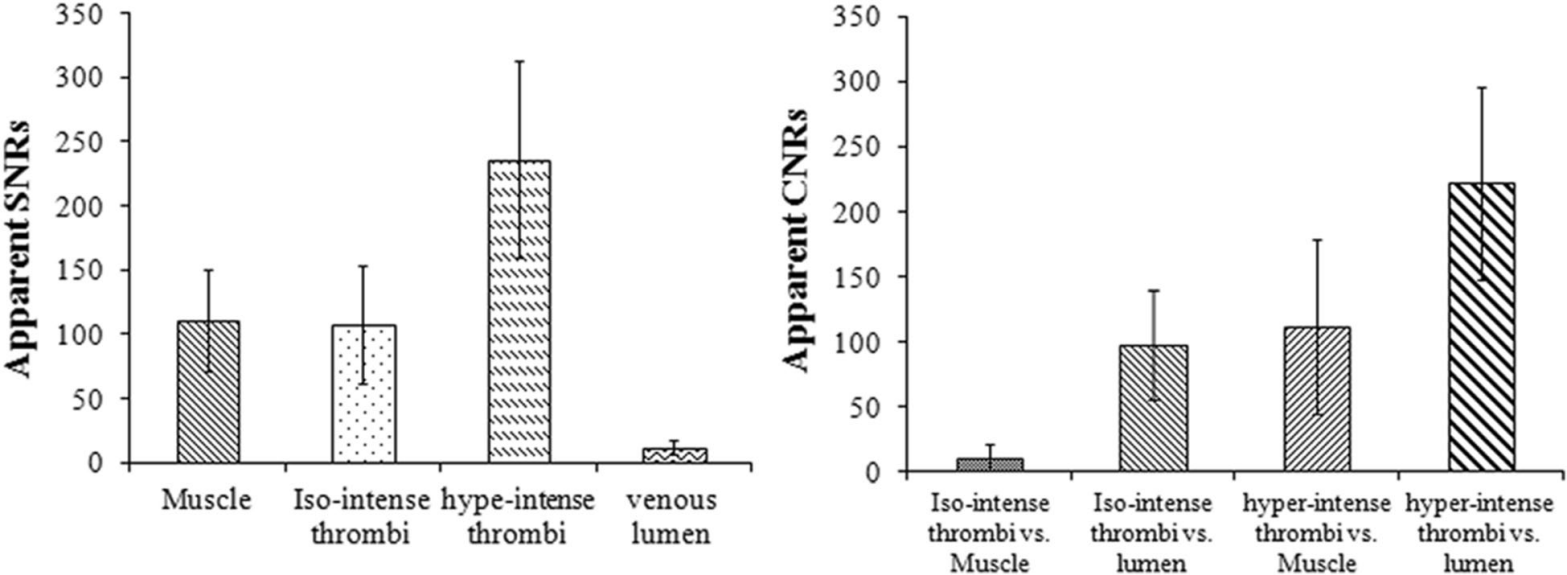
Results of quantitative signal to noise (SNR) and contrast to noise (CNR) analysis. Both iso−/hyperintense thrombi had adequate signal intensity and contrast to the black-blood venous lumen for the diagnosis of DVT

**Fig. 5:**
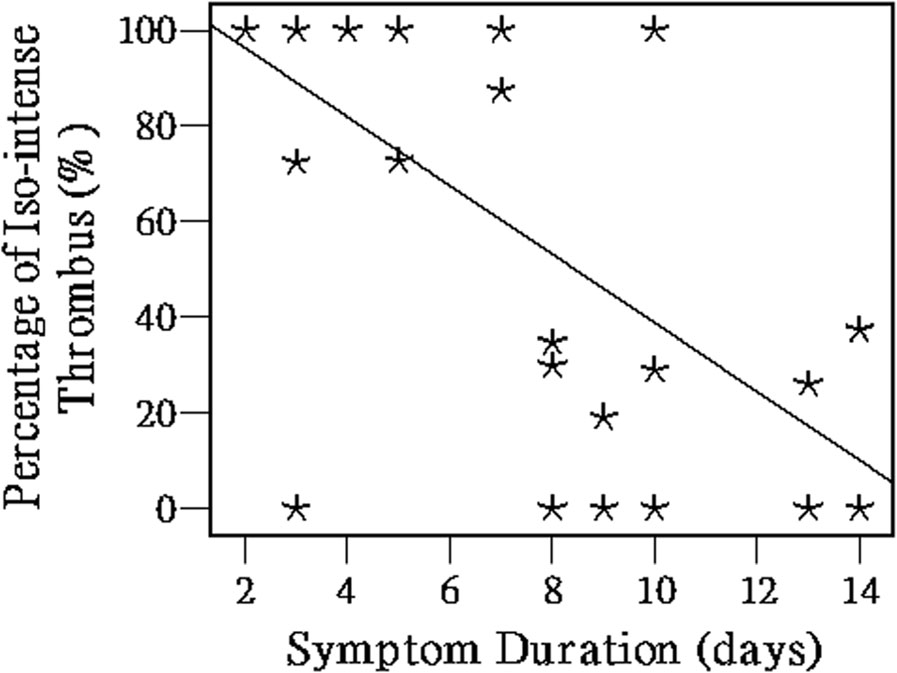
Volume percentage of the iso-intense thrombus in total thrombus volume of each patient. According to the linear regression analysis, the volume percentage of the iso-intense thrombus decreases with the duration of symptoms, indicating that the thrombus signal intensity tends to become stronger

**Fig. 6:**
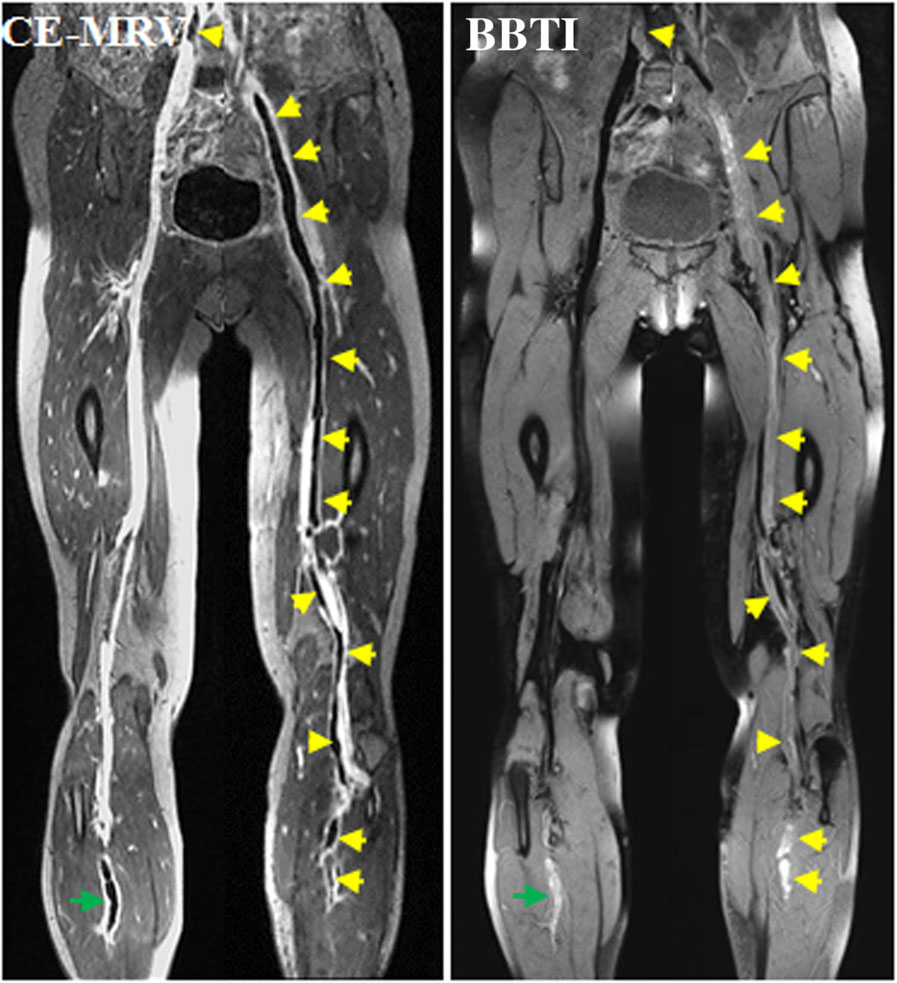
Example images obtained in 50-year-old woman with DVT symptom onset at 3 days. The thrombus (green arrows) was detected by BBTI at the asymptomatic right leg, which was missed by the initial ultrasound and confirmed by CE-CMRV. Most parts of the thrombi (yellow arrows) appeared as isointense signals on the BBTI images. The locations and sizes of the thrombi between BBTI and CE-CMRV matched

All participants successfully completed the CMR examinations, and no adverse events occurred. The thrombi within the black-blood venous lumen appeared iso- and/or hyper-intense relative to the muscle signal intensity on the BBTI images. Both large thrombi (Figs. 1 and 2) and small thrombi (Fig. 3) were correctly visualized by BBTI. Their location and size were visually matched with those detected using CE-CMRV. According to the apparent SNR and CNR analysis, both the iso- and hyperintense thrombi had adequate signal intensities for diagnosis (Fig. 4). According to the linear regression analysis, the thrombus signal intensity has the tendency to become higher as the duration of symptom onset increases (Figs. 5 and 6).

The image quality (3.62 ± 0.50 vs. 3.62 ± 0.59) and diagnostic confidence (3.68 ± 0.48 vs. 3.67 ± 0.56) of BBTI were good or excellent for the healthy subjects and patients. No part of any venous segment from the healthy subjects was misidentified as being positive by either reader. In other words, all the healthy subjecs were correctly identified from among the participants by both readers, using only the BBTI images. The interobserver agreements of BBTI were excellent in all the venous segments of the healthy subjects and patients regarding the presence or absence of thrombus, image quality, and diagnostic confidence (Table 3).

**Table 3:** Results of interobserver agreement between the two readers for diagnosis of DVT, image quality, and diagnostic confidence of BBTI

	Diagnosis of DVT	Image Quality	Diagnostic Confidence
Vessel segments	Interobserver agreement (**(**κ / *p***)**)	Interobserver agreement (**(**κ / *p***)**)	Interobserver agreement (**(**κ / *p***)**)
Abdominopelvic veins	1.00 / < 0.001	0.78 / < 0.001	0.61 / < 0.001
Femoral-popliteal veins	0.95 / < 0.001	0.69 / < 0.001	0.65 / < 0.001
Calf veins	0.87 / < 0.001	0.73 / < 0.001	0.75 / < 0.001
Superficial veins	0.88 / *<* 0.001	0.79 / < 0.001	0.77 / < 0.001
Collateral branch veins	0.91 / < 0.001	0.95 / < 0.001	0.83 / < 0.001
Overall	0.94 / < 0.001	0.82 / < 0.001	0.80 / < 0.001

Note: *DVT* deep vein thrombosis, *BBTI* black-blood magnetic resonance thrombus imaging

In total, 870 venous segments from thirty patients were observed by both BBTI and CE-CMRV. Eight of these (i.e., one popliteal vein, 2 anterior tibial veins, 1 posterior tibial veins, 3 fibular veins, and 1 small saphenous vein) demonstrated poor diagnostic confidence (i.e., score < 2) on the BBTI images and three (i.e., one popliteal vein, 1 posterior tibial vein, and 1 fibular vein) were seen on the CE-CMRV images. Thus, these eight segments were excluded, and the other 862 segments were used for statistical analysis. BBTI provided a comparable diagnostic confidence score for patients compared with CE-CMRV (average over the two readers: 3.69 ± 0.52 vs. 3.70 ± 0.47, *p* = 0.23). All the patients were confirmed to have thrombi on both the BBTI and CE-CMRV images. According to the consensus reading of CE-CMRV, thrombi were identified in 227 of 862 venous segments (26.3%) (Table 4). No free-floating thrombus was detected by either BBTI or CE-CMRV. The underlying reason may be related to the limits of spatial resolution and the lack of dynamic information provided by BBTI and CE-CMRV. The locations of the thrombi in the 227 venous segments included 65 abdominopelvic venous segments (28.6%), 92 femoral-popliteal venous segments (40.5%), 43 calf venous segments (18.9%), 9 superficial venous segments (4.0%), and 18 collateral branch venous segments (7.9%). Both CE-CMRV and BBTI showed above-knee DVT in 8 patients, calf DVT associated with above-knee DVT in 22 patients, and 8 of the 30 patients had IVC thrombosis. Using the consensus CE-CMRV diagnosis as the reference standard, the overall sensitivity (95.2%), SP (98.6%), positive predictive value (96.0%), negative predictive value (98.3%), and accuracy (97.7%) were obtained by BBTI (Table 5). In addition, BBTI had a higher sensitivity (97.4 vs. 81.4%), specificity (99.3 vs. 99.0%), and accuracy (98.7 vs. 95.7%) for the diagnosis of DVT above the knee compared to that below the knee. Notably, thrombus was also identified in the asymptomatic legs of three patients using BBTI; this was not identified by the initial ultrasound test and confirmed by CE-CMRV (Fig. 6).

**Table 4:** Results of BBTI and CE-CMRV examinations in 862 vessel segments

Vessel segments	BBTI(Reader1/Reader2)		Consensus of CE-CMRV	
	Thrombus present	Thrombus absent	Thrombus present	Thrombus absent
Abdominopelvic veins				
Inferior vena cava	8/8	22/22	8	22
Common iliac vein	18/18	42/42	18	42
Internal iliac vein	16/16	44/44	18	42
External iliac vein	21/21	39/39	21	39
Femoral-popliteal veins				
Common femoral vein	23/24	37/36	24	36
Femoral vein	27/27	33/33	27	33
Deep femoral vein	17/15	43/45	16	44
Popliteal vein	27/25	32/34	25	34
Calf veins				
Tibiofibular trunk vein	14/17	46/43	16	44
Anterior tibial vein	1/2	57/56	3	55
Posterior tibial vein	11/11	48/48	11	48
Fibular vein	11/8	46/49	13	44
Superficial veins				
Great saphenous vein	9/8	51/52	9	51
Small saphenous vein	0/1	59/58	0	59
Collateral branch veins	22/21	38/39	18	42
Total	225/222	637/640	227	635

Note: *BBTI* black-blood magnetic resonance thrombus imaging, *CE-CMRV* contrast-enhanced cardiovascular magnetic resonance venography

**Table 5:** Qualitative and statistical analysis results of BBTI for the diagnosis of DVT using consensus CE-CMRV as the reference standard

Vessel segments	Sensitivity (%) Reader1 /Reader 2	Specificity (%) Reader1 /Reader 2	Positive predictive value (%) Reader1 /Reader 2	Negative predictive value (%) Reader1 /Reader 2	Accuracy (%) Reader1 /Reader 2
Abdominopelvic veins	98.5 / 98.5	100 / 100	100 / 100	99.3 / 99.3	99.5 / 99.5
Femoral-popliteal veins	98.9 / 96.7	98.0 / 98.6	96.8 / 97.8	99.3 / 98.0	98.3 / 97.9
Calf veins	81.4 / 79.1	99.0 / 97.9	94.6 / 89.5	95.9 / 95.4	95.7 / 94.4
The superficial veins	100 / 88.9	100 / 99.1	100 / 88.9	100 / 99.1	100 / 98.3
Collateral branch veins	100 / 94.4	90.5 / 90.5	81.8 / 81.0	100 / 97.4	93.3 / 91.7
Overall	95.2 / 93.0	98.6 / 98.3	96.0 / 95.0	98.3 / 97.5	97.7 / 96.9

Note: *BBTI* black-blood magnetic resonance thrombus imaging, *CE-CMRV* contrast-enhanced cardiovascular magnetic resonance venography

Excellent agreement was noted between BBTI and CE-CMRV regarding the presence or absence of thrombus (reader 1, κ = 0.94, *p* <  0.001; and reader 2, κ = 0.92, *p* <  0.001).

## Discussion

In this prospective study, BBTI demonstrated a high sensitivity, specificity, and accuracy for the diagnosis of acute DVT at 1.5 T, without the use of a CMR contrast agent. Additionally, excellent diagnostic confidence scores and agreement with CE-CMRV on the diagnosis of DVT were achieved by BBTI. These findings were consistent with those of a previous study at 3 T [23] Thus, BBTI has the capacity to diagnose acute DVT at 1.5 T.

BBTI has some remarkable technical advantages for the diagnosis of DVT. If the venous blood flow signals are effectively suppressed while the thrombus has adequate signal intensity, the thrombus can be directly identified within the venous lumen. BBTI not only exploits the inherent black-blood effect of the SPACE readout but also the DANTE black-blood preparation for a more robust signal suppression of venous blood flow [28, 29]. Additionally, the SPACE readout used in BBTI is a variant of a spin echo sequence for 3D imaging that has an intrinsically high SNR and rapid data acquisition, which allows for the BBTI scan within a reasonable scan duration (i.e., less than 15 min for the entire lower limbs) and provides adequate thrombus signals for detection, even if the scan is performed at 1.5 T. Moreover, SPACE was configured as a T1-weighted readout. Because the image weighting of a DANTE sequence is dominated by the readout (not the DANTE preparation), [30, 31] BBTI is a T1-weighted technique that has advantages for the diagnosis of DVT. Thus, the diagnostic confidence score of BBTI is comparable to that of CE-CMRV, and an overall high sensitivity, specificity, negative/positive predictive value and accuracy can be obtained by BBTI at 1.5 T for the diagnosis of acute DVT.

BBTI may have a specific role in several scenarios for the diagnosis of DVT. First, unlike ultrasound, which often exclusively scans the symptomatic leg(s) with the goal of time efficiency, [32, 33] BBTI with a 3-station scan can cover both lower limbs. This feature is important for the diagnosis of asymptomatic DVT. In this study, DVT was detected in the asymptomatic contralateral legs of three patients, lesions that were not identified in the initial ultrasound test. Second, BBTI may be useful for the identification of thrombus in the pelvis, which is challenging for ultrasound [34]. In this study, a high sensitivity (98.5%) and specificity (100%) in the abdominopelvic segments without the use of a gadolinium based contrast agent were achieved using BBTI. Third, unlike the time-of flight, phase contrast, and bSSFP techniques, which indirectly identify the thrombus through blood filling defects, BBTI allows the direct visualization of the thrombus and measurement of thrombus signal intensity. This is beneficial for DVT treatment decision-making and could be complimentary to time of flight, phase contrast, and bSSFP imaging. Lastly, BBTI is more suited than ultrasound for the differentiation of acutely formed thrombus compared with chronic thrombus. The recurrence rate of DVT remains high (20–40%) [21, 35]. It is necessary to identify newly formed thrombus during monitoring of the progression of DVT and guide therapy for avoiding further complications such as pulmonary embolism [36]. As a noninvasive, contrast-free and time-efficient technique, BBTI could be suitable for this evaluation.

Signal variation of the thrombus in the acute phase was observed on the BBTI images. The thrombus signal change has the tendency to increase with the duration of symptom onset. This signal variation may result from variation in the concentration of methemoglobin in the thrombus. Previous studies have demonstrated that T1-weighted contrast is superior in the detection of thrombus because there is a linear relationship between the concentration of methemoglobin and T1 shortening of the venous thrombus [19, 37]. The higher the concentration of methemoglobin, the shorter the T1 relaxation time of the thrombus and thus, the brighter the thrombus signals on the T1-weighted image. BBTI may serve as a useful imaging tool for understanding thrombus progression because BBTI is a T1-weighed imaging technique and has the capacity for excellent separation between DVT and the surrounding venous lumen. This should be evaluated in a systematic study with a larger patient cohort.

BBTI provides a higher sensitivity above the knee than below the knee in acute DVT patients. Only two thrombi presenting as isointense in the internal iliac vein went unrecognized by either reader. However, a reassessment of the misinterpreted venous segments above the knee showed consistent results with CE-CMRV. Interpretation of the thrombus below the knee is more difficult. This may be caused by the small and complex anatomy of the calf vessel, the insufficient spatial resolution provided by BBTI, and the thrombus appearing as isointense relative to the adjacent muscle signals. Improving the spatial resolution is possible but would prolong the scan time and reduce the image SNR. Nevertheless, the interobserver agreement was excellent, and the findings in this study were consistent with current CMR techniques and ultrasound [18, 32, 38, 39].

It should be noted that some residual blood signals remain apparent on the BBTI images because there is little or no blood flow. This is not a problem unique to the BBTI technique, but is a common problem when the contrast is based on blood flow suppression or enhancement, such as with the widely used technique of ultrasound. Nevertheless, the thrombus and residual blood can be differentiated by an experienced radiologist. This is because DVT is often characterized by obstruction/dilatation of the involved veins and the clot is heterogeneous. Thus, the thrombus appeared as a signal with isolated and/or inhomogeneity intensity within the black venous lumen. In contrast, the residual blood often appeared as an iso- and homogeneous intense signal at the tortuous popliteal vein and the small calf vein.

There were several limitations to this work. First, the leg fat on the BBTI images was not suppressed well due to B0/B1 field inhomogeneity. However, the thrombus was within the venous lumen and the BBTI images could be reformatted with arbitrary orientation. Both the qualitative and quantitative analysis results demonstrated that fat suppression was not a significant issue in the identification of the thrombus. Second, conventional x-ray venography was not available for comparison as the reference standard. That for our study was established by consensus with CE-CMRV and, if necessary, with the assistance of ultrasound. This is because conventional x-ray venography is rarely used as a purely diagnostic modality in clinical settings. CE-CMRV has been shown in multiple studies to have an extremely high sensitivity and specificity compared with contrast venography [12, 14, 34]. Third, gadopentetate dimeglumine was used as the contrast agent because it is a globally recognized agent with a history of wide use, and it possesses the most comprehensive safety database. However, gadobenate dimeglumine could be considered for as a contrast agent in future studies because it can achieve the same contrast effect at a lower dose. Fourth, the current data only focused on acute DVT patients. Further evaluation of the diagnostic performance for thrombus at different phases should be performed on a larger group of DVT patients in future studies.

## Conclusions

BBTI exhibits excellent diagnostic performance on acute DVT at 1.5 T. BBTI is a promising technique that can be used for the diagnosis in clinical practice of acute DVT without the use of a contrast agent.

## Abbreviations

3D: Three-dimensional
BBTI: Black-blood thrombus imaging
bSSFP: Balanced steady state free precession
CE-CMRV: Contrast-enhanced cardiovascular magnetic resonance venography
CMR: Cardiovascular magnetic Resonance
CNR: Contrast-to-noise ratio
DANTE: Delay alternating with nutation for tailored excitation
DVT: Deep vein thrombosis
FLASH: Fast low-angle shot
IVC: Inferior vena cava
MRDTI: Magnetic resonance direct thrombus imaging
PTS: Post-thrombotic syndrome
ROI: Region-of-interest
SD: Standard deviation
SI: Signal intensity
SNR: Signal-to-noise ratio
SPACE: three-dimensional variable flip angle turbo-spin-echo
T: Tesla
